# Molecular probes and switches for functional analysis of receptors, ion channels and synaptic networks

**DOI:** 10.3389/fnmol.2013.00048

**Published:** 2013-12-13

**Authors:** Pau Gorostiza, Daniele Arosio, Piotr Bregestovski

**Affiliations:** ^1^ICREA and Institute for Bioengineering of CataloniaBarcelona, Spain; ^2^Institute of Biophysics, National Research Council and FBKTrento, Italy; ^3^Inserm UMR1106, Brain Dynamics Institute, University Aix-MarseilleMarseille, France

**Keywords:** biosensors, chloride imaging, pH monitoring, optogenetics, optopharmacology, photochromic ligands

Photochromic switches and genetically encoded biosensors have become powerful tools for monitoring and modulating the activity of neurons and neuronal networks. Our understanding of the mechanisms underlying the development and functioning of the nervous system has greatly advanced in recent years thanks to advancements in these effective molecular and genetic tools. The idea of this Special Research Issue of *Frontiers in Molecular Neuroscience* is to provide an overview of the approaches in this area of research, and to present new applications in molecular imaging of ions and remote activation of receptors, ionic channels and synaptic networks. The issue contains experimental and methodological papers as well as review articles dealing with molecular tools for investigation and modulation of neuronal function. It can be divided into two main sections: (i) genetically encoded probes for non-invasive monitoring of ions and ATP; and (ii) optogenetic and optopharmacologic tools for control of neuronal activity with light.

In the first section, several papers are devoted to the use of probes for the non-invasive monitoring of intracellular chloride ([Cl^−^]_i_). A sensor with improved sensitivity to chloride, called Cl-Sensor (Markova et al., 2008), has previously been used to measure [Cl^−^]_i_ in different cell types and to analyse the function of the potassium-chloride transporter KCC2 in hippocampal neurons (Bregestovski et al., 2009; Waseem et al., 2010; Pellegrino et al., 2011). In this issue, improved methods for the stable, long-lasting ratiometric recording of [Cl^−^]_i_ are described, and these provide a technique for monitoring Cl-Sensor fluorescence in different cell types using conventional fluorescence microscopy set-ups (Friedel et al., 2013). Cl-Sensor has also been used to analyse the mechanisms leading to changes in neuronal [Cl^−^]_i_ during glioma invasion (Bertollini et al., 2012). The authors demonstrated that glioma cells induce release of amino acids, which may dynamically alter Cl^−^ equilibrium in surrounding neurons. This causes interference with their inhibitory balance, probably leading to physiological and pathological consequences (Bertollini et al., 2012). An important development is the production of two mouse lines expressing Cl-Sensor, which allows ratiometric monitoring of [Cl^−^]_i_ in specific cell types *in vivo* (Batti et al., 2013).

Recently, a probe allowing simultaneous monitoring of Cl^−^ and H^+^ has been developed (Arosio et al., 2010). In this issue, a study reports the intracellular calibration and functional characterization of this sensor, called ClopHensor, and its two derivatives: the membrane-targeting PalmPalm-ClopHensor and the H148G/V224L mutant with improved Cl^−^ affinity and reduced pH dependence (Mukhtarov et al., 2013). This study identified the different ClopHensor variants as promising tools for non-invasive measurement of [Cl^−^]_i_ and pH in living cells. The usefulness of GFP-derived pH reporters to quantify intracellular pH in the context of changing neuronal activity is demonstrated in another study, where the authors compare three genetically encoded probes to analyse pH transients evoked by epileptiform activity in two separate *in vitro* models of temporal lobe epilepsy (Raimondo et al., 2012).

Two papers illustrate the use of genetically encoded probes to analyse physiologically important ions or molecules in different cellular compartments (Surin et al., 2012; Akerboom et al., 2013). The first study describes a family of genetically encoded calcium indicators. This engineered set of chromatic variants facilitates experiments in functional imaging and optogenetics and allows the simultaneous monitoring of intracellular calcium ([Ca^2+^]_i_) in different cell types (i.e., neurons and astrocytes) or cellular compartments (i.e., cytoplasm and mitochondria) (Akerboom et al., 2013). The second contribution presents a comparative analysis of cytosolic and mitochondrial ATP synthesis in embryonic and postnatal hippocampal neuronal cultures (Surin et al., 2012). The authors simultaneously monitored ATP and mitochondrial membrane potential using a genetically encoded sensor (Imamura et al., 2009). Their observations suggest that ATP synthesis is predominantly glycolytic in embryonic but not in postnatal neuronal cultures.

The last article in the first section (Brondi et al., 2012) describes a method for combining calcium imaging and genetic labeling of specific cell types in the mouse brain, using fluorophores with very similar emission spectra. The authors exploit the differences in two-photon emission spectra of the dyes and demonstrate that this technique can be extended to other fluorophores.

The second section of this Special Research Issue presents optogenetic tools and photoswitches for the optical control of neuronal activity. A method for studying neuronal plasticity following long-term neuronal stimulation using Channelrhodopsin-2 and monitoring by multi-electrode arrays is presented by Lignani et al. (2013). Optogenetics can also be used to study the molecular mechanisms associated with drugs of abuse (Chandra et al., 2013). The authors show that altering the activity in specific neural circuits can result in the regulation of cocaine-induced actin-cytoskeleton dynamics and behavioral plasticity.

The fields of optopharmacology and optochemical genetics are reviewed by Sandoz and Levitz (2013) and Mourot et al. (2013) with special emphasis on their application to neuronal potassium channels. Ion channels and receptors can be photosensitized using synthetic light-regulated ligands that either diffuse freely or are permanently conjugated to their target channel. Light acting on the ligands allows their reversible binding to ion channels, leading to activation or inhibition depending on the nature of the ligand. These methods provide powerful biophysical tools for fundamental studies of ion channels, and promising therapeutic applications such as the photosensitization of blind retinas. The study by Izquierdo-Serra et al. (2013) applies calcium photocurrents through a light-gated glutamate receptor to control calcium-regulated exocytosis and optically modulate neurotransmission.

The issue concludes with two articles that are focused on the approaches and techniques to analyse Cl-selective receptor-operated channels. Lynagh and Lynch (2012) used a voltage-clamp fluorometry to quantitatively monitor agonist-induced conformational and fluorescence changes at locations distant from the ionic pore. In this way, they analyzed the molecular mechanisms of cys-loop channel modulation by ivermectin. The review by Schaefer et al. (2012) describes mouse models in combination with modern imaging techniques as excellent tools for bridging the gap between adaptation and remodeling of inhibitory synapses under physiological and pathological conditions.

We are grateful to all authors and reviewers for their efforts in providing an excellent overview of these rapidly expanding fields.
